# Aldehyde dehydrogenase 1A1 in stem cells and cancer

**DOI:** 10.18632/oncotarget.6920

**Published:** 2016-01-15

**Authors:** Hiroyuki Tomita, Kaori Tanaka, Takuji Tanaka, Akira Hara

**Affiliations:** ^1^ Department of Tumor Pathology, Gifu University Graduate School of Medicine, Gifu, Japan; ^2^ Department of Surgical Oncology, Gifu University Graduate School of Medicine, Gifu, Japan; ^3^ Division of Pathology, Gifu Municipal Hospital, Gifu, Japan

**Keywords:** aldehyde dehydrogenase, ALDH, ALDH1A1, cancer, stem cell

## Abstract

The human genome contains 19 putatively functional aldehyde dehydrogenase (ALDH) genes, which encode enzymes critical for detoxification of endogenous and exogenous aldehyde substrates through NAD(P)^+^-dependent oxidation. ALDH1 has three main isotypes, ALDH1A1, ALDH1A2, and ALDH1A3, and is a marker of normal tissue stem cells (SC) and cancer stem cells (CSC), where it is involved in self-renewal, differentiation and self-protection. Experiments with murine and human cells indicate that ALDH1 activity, predominantly attributed to isotype ALDH1A1, is tissue- and cancer-specific. High ALDH1 activity and ALDH1A1 overexpression are associated with poor cancer prognosis, though high ALDH1 and ALDH1A1 levels do not always correlate with highly malignant phenotypes and poor clinical outcome. In cancer therapy, ALDH1A1 provides a useful therapeutic CSC target in tissue types that normally do not express high levels of ALDH1A1, including breast, lung, esophagus, colon and stomach. Here we review the functions and mechanisms of ALDH1A1, the key ALDH isozyme linked to SC populations and an important contributor to CSC function in cancers, and we outline its potential in future anticancer strategies.

## INTRODUCTION

Aldehyde dehydrogenases (ALDHs) are a group of nicotinamide-adenine dinucleotide phosphate–positive (NAD(P)^+^)-dependent enzymes that catalyze the oxidation of endogenous and exogenous aldehyde substrates to their corresponding carboxylic acids [1-3]. Endogenous aldehydes are generated during the metabolism of amino acids, alcohols, lipids, and vitamins, while exogenous aldehydes, as intermediates or products, are derived from the metabolism of a wide variety of environmental agents and drugs (e.g., cigarette smoke, vehicle exhaust fumes and cytotoxic drugs) [2]. The enzymes show various biological functions, including cellular detoxification. More than 160 ALDH cDNAs or genes have been isolated and sequenced from various sources (e.g., bacteria, yeast, fungi, plants and animals), and studies report 19 putatively functional genes and many pseudogenes in the human genome (Table 1) [4].

**Table 1:** Human ALDH isoenzymes

Isoenzymes	prefered substrates	Subcellular distribution	Organ and tissue distribution	Chromosomal locaization
ALDH1A1	Retinal	Cytosol	Liver, kidney, red blood cells, skeletal muscle, lung, breast, lens, stomach, brain, pancreas, testis, prostate, ovary	9q21,13
ALDH1A2	Retinal	Cytosol	Testis, liver, kidney	15q21.3
ALDH1A3	Retinal	Cytosol	Kidney, skeletal muscle, lung, breast, stomach, salivary glands	15q21.3
ALDH1B1	Acetaldehyde, lipid peroxidation-derived aldehdes	Mitochondria	Liver, kidney, heart, skeletal muscle, brain, prostate, lung, teastis, placenta	9p11,1
ALDH1L1	10-Formyltetrahydrofolate	Cytosol	Liver, skeltal muscle, kidney	3q21.3
ALDH1L2	Unknown	Cytosol		12q23.3
ALDH2	Acetaldehyde, nitroglycerin	Mitochondria	Liver, kidney, heart, skeletal muscle, lens, brain, pancreas, prostate, spleen	12q24.2
ALDH3A1	Medium-chain aliphatic and aromatic aldehydes	Cytosol, nucleus	Stomach, cornea, breast, lung, lens, esophagus, salivary glands, skin	17p11.2
ALDH3A2	Long-chain aliphatic aldehydes	Microsomes, peroxisomes	Liver, kidney, heart, skeletal muscle, lung, brain, pancreas, placenta, most tissues	17p11.2
ALDH3B1	Lipid peroxidation-derived aldehydes	Mitochondria	Kidney, lung, pancreas, placenta	11q13
ALDH3B2	Unknown	Mitochondria	Parotid gland	11q13
ALDH4A1	Proline metabolism	Mitochondria	Liver, kidney, heart, skeletal muscle, brain, pancreas, placenta, lung, spleen	1p36
ALDH5A1	Succinic semialdehyde	Mitochondria	Liver, kidney, heart, skeletal muscle, brain	6p22
ALDH6A1	Methylmalonate semialdehyde	Mitochondria	Liver, kidney, heart, skeletal muscle	14q24.3
ALDH7A1	Betane aldehyde, lipid peroxidation-derived aldehydes	Mitochondria, nucleus, cytosol	Fetal liver, kidney, heart, lung, brain, ovary, eye, cochlea, spleen adult spinal cord	5q31
ALDH8A1	Retinal	Cytosol	Liver, kidney, brain, breast, testis	6q23.2
ALDH9A1	γ-Aminobutyraldehyde, aminoaldehydes	Cytosol	Liver, kidney, heart, skeletal muscle, brain, pancreas, adrenal gland, spinal cord	1q23.1
ALDH16A1	Unknown	Unkown	Neuronal cells	19q13.33
ALDH18A1	Glutamatic γ-semialdehyde	Mitochondria	Kidney, heart, skeletal muscle, pancreas, testis, prostate, spleen,ovary, thymus	10q24.3

ALDH1 proteins (mainly ALDH1A1, ALDH1A2 and ALDH1A3), which are primarily localized in the cytosol of cells from various tissues, include enzymes able to oxidize retinal and aliphatic aldehydes. They exhibit high activity for oxidation of aldophosphamide and play a role in the detoxification of some commonly used anticancer drugs, such as oxazaphosphorines [5, 6]. They also play a role in the detoxification of peroxidic aldehydes produced by ultraviolet light absorption, protecting the lens of the eye [7]. Moreover, it has been demonstrated that cancer cell-acquired drug resistance is associated with the transcriptional activation of ALDH1 expression [8].

*ALDH1A1* encodes a homotetramer that is ubiquitously distributed in adult organs, such as brain, testis, kidney, eye, lens, retina, liver, and lungs. ALDH1A1 takes its position among the three highly conserved cytosolic isozymes (ALDH1A2 and ALDH1A3), which catalyze the oxidation of retinal (retinaldehyde), the retinol metabolite, to retinoic acid (RA). ALDH1A1 has great affinity for the oxidation of both all-trans- and 9-cis-retinal. By serving as a ligand for nuclear RA receptors (RARs) and retinoid X receptors (RXRs), RA regulates gene expression; therefore, its synthesis is crucial for normal growth, differentiation, development, and maintenance of adult organs and tissues in vertebrate animals.

Historically, ALDH1A1 has been the key ALDH isozyme linked to stem cell (SC) populations. ALDH1A1 also plays a vital role as a marker of SCs and cancer stem cells (CSCs). Despite accumulating evidence on the functional role of ALDH1A1 in SCs and CSCs, the specific mechanisms involved in the regulation of ALDH1A1 in SCs and CSCs remain unclear. Thus, this review focuses on the biological and functional effects and mechanisms of ALDH1A1, which is an isotype of ALDH1, and the mechanisms underlying ALDH1A1 regulation in SCs and CSCs, and provides insights into the potential therapeutic applications of ALDH1A1 in CSC elimination from cancer tissues.

## THE BIOLOGICAL AND FUNCTIONAL MECHANISMS OF ALDH1A1

**Figure 1:**
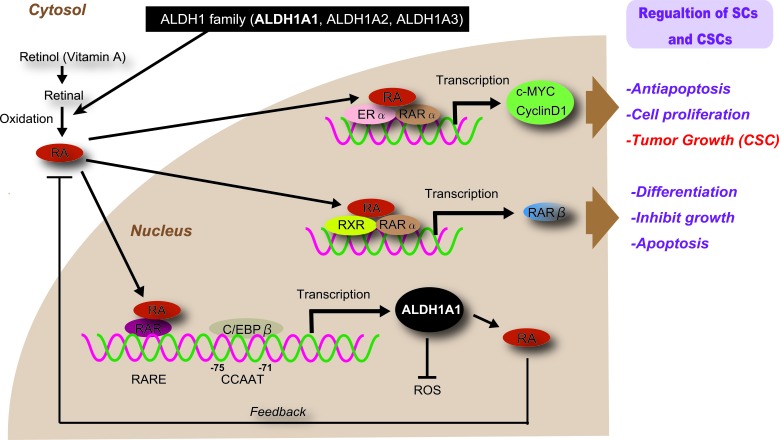
Regulation and function of ALDH1 in normal SCs and CSCs Several ALDHs metabolize RA, thereby regulating the self-renewal, differentiation, and tumor resistance of SCs and CSCs. Retinol absorbed by cells is oxidized to retinal. Retinal is oxidized to RA by ALDH1 enzymes. RA binds to dimers of RARα and RXRs to induce the expression of its downstream target genes including RARβ. In ERα-expressing cells, RA can bind to dimers of RXRs and ERα as well as induce the expression of c-MYC and cyclinD1. RA, Retinoic acid; RAR, Retinoic acid receptor; RXR, retinoid X receptors; ER, Estrogen receptor; ROS, Reactive oxygen species.

**Figure 2:**
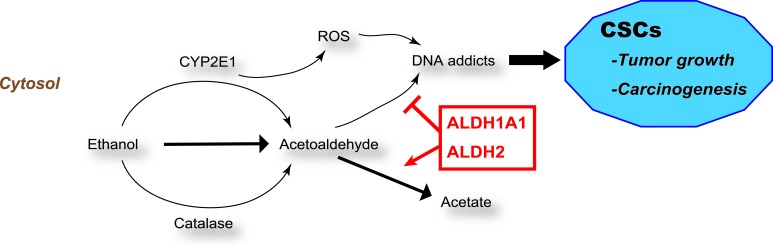
ALDHs and ROS in carcinogenesis ALDHs reduce ROS and reactive aldehydes, thereby promoting tumor growth and initiating carcinogenesis in CSCs. ROS, Reactive oxygen species

The mechanisms underlying the effects of ALDHs in SC and CSC maintenance remain unclear. However, the regulated RA, reactive oxygen species (ROS), and reactive aldehyde metabolism are likely to be closely related with its functional roles (Figures 1 and 2).

### Role of ALDH1A1 in retinoid signaling

Retinoid signaling pathways play significant roles in embryonic stem cells [9] and cancer cells [10]. RA and its derivatives are involved in many critical physiological processes, including the regulation of gene expression, morphogenesis, and development [11-13]. In retinol metabolism (Figure 1), retinol dehydrogenases oxidize the retinol (vitamin A) absorbed by cells to retinal. Then, retinal is oxidized to RA in a reaction catalyzed by ALDH1A1, ALDH1A2, ALDH1A3, and ALDH8A1. The metabolized product RA includes all-trans RA (ATRA), 9-*cis* RA, and 13-*cis* RA. The ALDH isoforms, especially ALDH1A1, have affinity for ATRA and 9-*cis* RA. RA can enter the nucleus and induce the transcriptional activity of downstream effectors through the activation of heterodimers of RAR (RAR-α, β, γ) and RXR (RXR-α, β, γ).

ALDH1A1 promoter contains a positive regulatory region (−91 to +53) with a CCAAT box as a major *cis*-acting element [14]. When endogenous RA concentrations are low, the RAR binds to the retinoic acid response element (RARE), and the CCAAT/enhancer-binding protein-β (C/EBPβ) binds to the CCAAT box. Together, the RAR and C/EBPβtransactivate the *Aldh1* promoter, and activate transcription. Increasing ALDH1 levels can result in an increase in RA synthesis, as well as cellular protection against cytotoxic drugs.

For example, Ginestier *et al.* [15] have reported that ALDH1 regulates breast CSCs by affecting retinoid metabolism; retinoid signaling modulation may be sufficient to induce the differentiation of breast CSCs. RA can bind to RA and RX receptors and activate gene expression related to loss of SC markers, differentiation, cell cycle arrest, and morphology change [16]. The subsequent upregulation of these receptors generates a positive feedback loop for retinoid signaling. Currently, RA formation by oxidation of all-trans-retinal and 9-cis-retinal in retinoid signaling has been related to the “stemness” of both SCs and CSCs [17]. Thus, the functional role of ALDH1A1 in retinoid signaling is considered highly similar and extremely important for the regulation and maintenance of both SC and CSC.

### Role of ALDH1A1 in acetaldehyde metabolism

Ethanol is metabolized to acetaldehyde by alcohol dehydrogenase (ADH), catalase, and cytochrome P4502E1 (CYP2E1) (Figure 2). Acetaldehyde interferes with anti-oxidative defense systems and generates ROS. ROS inhibits DNA repair and methylation and forms DNA and protein adducts, promoting carcinogenesis and cancer growth [18-20]. Acetaldehyde is metabolized to acetate primarily by ALDH2 and ALDH1A1. ALDH activity is required to maintain sufficiently low ROS levels and prevent triggering CSC apoptosis [21]. ROS and reactive aldehydes metabolism are strongly related with various properties of CSC as well as tumor growth and carcinogenesis. However, the mechanisms that link ALDH to ROS in SCs and CSCs will require further research.

### ALDH1A1 and drug-resistance in chemotherapy

Given the reported functions of ALDH enzymes, it is not surprising that ALDHs are generally regarded as detoxification enzymes critical for protecting organisms against various harmful aldehydes [22-24]. ALDH1A1 and ALDH3A1 can offer cellular protection against cytotoxic drugs. It was first observed over two decades ago that hematopoietic and leukemic stem cells with ALDH activity were highly resistant to cyclophosphamide, an alkylating agent [6, 25]. Cytosolic ALDH1A1 and ALDH2A1 convert activated cyclophosphamide, 4-hydroperoxycyclophosphamide, to the inactive excretory product carboxyphosphamide [25, 26]. Thus, ALDH1A1 can provide drug protection and radiation resistance to CSCs.

## ALDH1A1 IN NORMAL TISSUE SCS AND TISSUES

**Figure 3:**
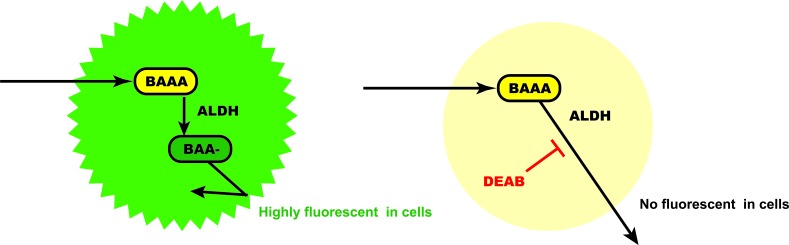
Scheme of the Aldeflour assay ALDH converts the ALDH substrate, BAAA(BODIPY-aminoacetaldehyde), into the fluorescent product BAA-(BODIPY-aminoacetate), which is retained inside viable cells. DEAB, diethylaminobenzaldehyde

It has been demonstrated that ALDH1 family members are strongly active in normal tissue SCs. Therefore, ALDH may be considered a marker for SCs and play a functional role, in terms of self-protection, differentiation, and expansion of SC populations. Furthermore, there are several isoforms of ALDH (ALDH1A1, ALDH1A2, ALDH1A3, and ALDH8A1) that play a role in RA formation by oxidation of all-trans-retinal and 9-cis-retinal, which are involved in retinoid signaling and have been related to the stemness of SCs [17]. These observations have been facilitated by the ability to functionally assess ALDH activity in live cells (Aldefluor assay kit, StemCell Technologies, Durham, NC, USA). For example, Ginestier *et al.* [27] masterfully overcame the toxicity associated with the Hoechst 33342 dye by using the Aldefluor assay, optimized to detect ALDH activity. The Aldefluor assay detects intracellular ALDH expression in viable cells through use of the green fluorescence channel of a standard flow cytometer (Figure 3). Because Aldefluor-positive cells are viable and selectable using flow cytometric cell sorting based on their fluorescence, they are readily available for both *in vitro* and *in vivo* SC studies. Importantly, the high ALDH activity in the Aldefluor assay has been attributed to ALDH1 expression [28], however, the assay cannot separate ALDH1 isotype-specific expression, i.e. ALDH1A1, ALDH1A2, or ALDH1A3.

Although ALDH1A1 was first indicated as a marker and a characteristic feature of primitive human hematopoietic stem cells (HSCs) isolated from bone marrow [29] and of neural stem cells [30, 31], recent studies have reported that other ALDHs (i.e., ALDH1A2, ALDH1A3, ALDH1A7, ALDH2*2, ALDH3A1, ALDH4A1, ALDH5A1, ALDH6A1, and ALDH9A1 [32, 33]) are also involved, because ALDH1A1 deficiency does not alter Aldefluor positivity in the analysis of *Aldh1a1* −/− mouse. It is believed that this heterogeneity indicates that the isoform responsible for Aldefluor activity in the case of normal SCs depends on the type of cell (Table 2). That is why most studies on ALDHs and SCs do not specify the ALDH isoform, but refer rather generically to ALDH. Interestingly, it has been reported that ALDH1A1, a predominant isoform in mammalian tissues, may serve as an SC marker in somatic SCs by immunohistochemistry (IHC) with ALDH1A1 isotype-specific antibody because of the very small population of ALDH1A1 expressing cells [34, 35].

**Table 2:** ALDH1 expression in various normal stem/progenitor cells

Cell types	ALDH1 isozymes	References
Hematopoetic progenitor cells	ALDH1, ALDH1A1*	25, 29
Mesenchymal prognitor cells	ALDH1	29
Endothelial progenitor cells	ALDH1	29
Neural stem cells	ALDH, ALDH1L1	34, 38
Liver progenitor cells	ALDH1	69
Normal mammary stem cells	ALDH1	27, 44
Pancreatic progenitor cells	ALDH1	46
Stomach stem cells	ALDH1	47
Prostate progenitor cells	ALDH1	42
Myogenic cells	ALDH1	39, 40
Colon stem cells	ALDH1, ALDH1B1	43

* This data is based on Aldh1a1 knockout mouse.

The high ALDH activity in HSCs is associated with an enhanced regeneration and cellular resistance to cytotoxic drugs. Indeed, the presence of high ALDH activity makes HSCs resistant to alkylating agents, such as activated oxazaphosphorine (e.g., mafosfamide or 4-hydroperoxycyclophosphamide). Inhibiting ALDH activity with various inhibitors causes HSCs to become sensitive to these anticancer agents employed to purge resident tumor cells *ex vivo* or in the treatment of autologous bone marrow transplantation [26].

HSCs with high ALDH activity that have the ability to produce long-term multi-lineage hematopoietic colonies have been isolated from bone marrow, umbilical cord blood, and circulating cells [36]. As a result of their extreme brightness upon Aldefluor staining, cells with high ALDH activity have also been designated as ALDH-bright (ALDHbri) cell populations. The ALDHbri cell fraction ranges between 0.5% and 5.0% in human bone marrow, umbilical cord blood, and peripheral blood cells. The most intriguing recent observation is that ALDHbri populations isolated from bone marrow include hematopoietic, endothelial, mesenchymal, [29] and neural progenitor cells, which are crucial in repair protocols for various pathological conditions, such as ischemic diseases [37].

Several other types of normal tissue SCs display high levels of cytosolic ALDH1 expression: neural cells [38], myogenic cells [39, 40], mammary cells [41], prostate cells, [42] and intestinal crypt cells [43]. In mammary epithelial cells, Aldefluor positivity is predominantly due to high ALDH1 expression in positive cells, which account for 8% of the total population, and are able not only to self-renew, but also to generate both luminal and myoepithelial cells [44].

Recent reports on benign breast biopsies state that the ALDH1 activity associated with enhancer of zeste 2 polycomb repressive complex 2 subunit (EZH2), a protein involved in SC renewal and carcinogenesis, is a prognostic marker for the risk of developing cancer [45]. Furthermore, high ALDH1 activity, due to strong expression of both ALDH1A1 and ALDH1A7, has been identified in central-acinar/terminal duct cells from peripheral acinar duct units in studies carried out on murine pancreas [46]. ALDH1-positive cells (5%) are mainly located at the normal crypt bottom in the colon and stomach [43, 47]. In normal murine prostate, a cell subset expresses high levels of ALDH1 activity associated with antigens typical of prostate basal epithelium. When cells with low ALDH1 were compared with ALDH1-positive prostate cells, there was a 2.5-fold increase in the formation of prostatic tissue after *in vivo* transplantation [42]. Thus, ALDH1 including ALDH1A1 is expressed in various tissue SCs and normal tissues, although expression levels differ among each tissue.

## ALDH1A1 IN CSCS AND CANCER TISSUES

According to the recent “CSC” hypothesis, tumors contain a minor component of tumorigenic cells and a major component of non-tumorigenic cells [48, 49]. The minor population, termed CSCs or tumor-initiating cells, has pronounced tumorigenic activity in xenograft transplantation assays [50, 51]. Several isoforms (ALDH1A1, ALDH1A2, and ALDH1A3), play a role in RA formation through oxidation of all-trans-retinal and 9-cis-retinal that are involved in retinoid signaling, which has been related to the stemness of CSCs as well as normal tissue SCs [17, 32]. Furthermore, there is a relationship between ROS and high ALDH activity [52], and ALDH-high cells contain lower ROS levels than ALDH-low cells in certain malignancies, suggesting elevated antioxidant activity [53].

Recent reports implicate ALDH1 and specifically its isotype ALDH1A1 as a useful CSC marker that could be used to enrich tumor-initiating subpopulations from various cell lines and primary tumors [43, 44, 47]. Surprisingly, it has been documented that high ALDH1A1 expression does not always correlate with highly malignant phenotypes and poor clinical outcome in a range of cancers (Table 3). This result may depend on the method for detecting the ALDH1A1 expression [i.e., Aldefluor assay using flow cytometry or IHC with an ALDH1A1 antibody] and sample size or types of tissues [54, 55].

**Table 3:** ALDH1A1 overexpression in various cancer stem cells and cancer cell types

Tissue	Cancer type	Cancer stem cell	Prognosis	Assay	References
Liver	Hepatocellular carcinoma	×^a^ (differentiated hepatic cell)	Favorable	IHC^c^	68
	Hepatocellular carcinoma	×	Favorable	IHC	71
	Hepatocellular carcinoma	○^b^	-	ALDEFLUOR^d^	69, 70
Lung	Non-small cell carcinoma	-^e^	Poor	IHC	80
	Non-small cell carcinoma	○	Poor	IHC	55
	Non-small cell carcinoma	-	Favorable	IHC	81
Ovary	Adenocarcinoma	○ (drug resistance^f^)	-	ALDEFLUOR	84
	Adenocarcinoma	-	Favorable	IHC	86
Esophagus	Squamous cell carcinoma	○	Poor	IHC, ALDEFLUOR	87
Stomach	Adenocarcinoma	-	Poor	IHC	88
	Adenocarcinoma	-	No difference	IHC	89
Pancreas	Adenocarcinoma	○	Poor	IHC	90
	Adenocarcinoma	-	Favorable	IHC	91
Breast	Ductal carcinoma	○	Poor	IHC, ALDEFLUOR	27
	Ductal carcinoma	○	Poor	IHC	93
Colorectum	Adenocarcinoma	-	No difference	IHC	95
	Adenocarcinoma	○(drug resistance)	Poor	IHC	97

a “×” indicates not a cancer stem cell or differentiated cell.

b “○” indicates cancer stem cell.

c “IHC” indicates immunohistochemistry using the ALDH1A1 antibody.

d “ALDEFLUOR” indicates the Aldefluor assay using flow cytometry.

e “-” indicates no data.

f “drug resistance” indicates resistance to chemotherapy.

### The difference between normal tissue SCs and CSCs

Normal tissue SCs and CSCs share several mechanisms related to “stemness”, however, there are some differences between normal tissue SCs and CSCs. Currently, controversial issues have arisen regarding the differences between normal tissue SCs and CSCs [56]. The first is that, unlike the case of normal tissue SCs, which are usually oligo- or multipotent, it is unclear whether CSCs can give rise to multiple differentiated cell types. The second is that it is unclear whether normal cellular precursors of CSCs are, in fact, true normal tissue SCs; for example, the CSC model usually stands on the basis of experimental characterizations of cancer cell populations. Further, the elucidation of interactions between the local microenvironment, i.e., niche, and normal tissue SCs, is one of the main topics of SC research in normal tissue and cancers. For example, in normal SCs, the surrounding microenvironment is normal; however, CSCs usually have aberrant microenvironments, such as severe inflammation, hypoxia, and/or low nutrient conditions.

Human hematopoietic progenitor cells expand and delay differentiation *in vitro* after treatment with a chemical ALDH1 inhibitor, diethylaminobenzaldehyde (DEAB) [57]. ALDH1A1 overexpression in hematopoietic cells confers cyclophosphamide resistance [58]. As a result, ALDH1A1 may be a pivotal regulator of SC function and the main determinant of ALDH activity by Aldefluor assay in normal tissue SCs. On the other hand, the role of ALDH1A1 in CSCs was demonstrated in that ALDH1 high cells from breast cancer isolated by Aldeflour assay were the tumor-initiating cells, indicating ALDH1A1 might drive tumor proliferation, differentiation, and maintenance [27]. The differentiation and maturation of CSCs has been thought to be a cancer therapy for targeting CSCs. Thus, the role of ALDH1 in CSCs might not always coincide with that of normal tissue SCs.

### ALDH1A1 overexpression and high activity in various cancer types

Although the exact isoform of ALDH1 responsible for the enzyme activity assessed by BODIPY aminoacetaldehyde (BAAA) remains controversial [17, 59-61], ALDH1A1 is thought to have a predominant role [17]. In IHC analysis, ALDH1A1 can be specifically identified with isotype-specific antibodies. However, the more important and consistently used identifier of a SC population is the Aldefluor assay, which, although primarily dependent on ALDH1A1, also identifies ALDH1A2 and ALDH1A3 isotypes [62, 63]. Thus, much attention has been focused on the relationship between the expression of this isoform and the clinicopathologic parameters, including prognosis, of the various types of cancers.

Prognostic data on the ALDH1 in various cancers have been accumulated predominantly by using IHC of paraffin embedded cancer tissues with isotype-specific antibodies, ALDH1A1 or ALDH1A3. ALDH1A1 overexpression evaluated by IHC has been both correlated with both poor and favorable prognoses in various cancers (Table 3). On the contrary, experimental studies of ALDH activity of CSC have shown that ALDHbri cells separated by Aldeflour assay are more tumorigenic *in vitro* and *in vivo.* On the whole, the increase of ALDHbri cells is correlated with worse prognosis with a few exceptions, such as in melanoma [64, 65]. The reason for such contradiction is unclear, however it could be related to the cell origin of the cancer and degree of maturation [66, 67] On the other hand, IHC analysis of ALDH1A1 expression by isotype-specific antibody, such as ALDH1A1 antibody, have shown several cancer types have favorable prognoses. The differences could be associated with the maturation and differentiation of ALDH1A1 positive cells in cancers [68].

In fact, it is difficult to explain the discrepancies among the studies. A single cause could be the ALDH detection method (IHC versus Aldeflour assay) used, the type of tissue handling (paraffin-embedded versus fresh samples), cut-off levels of ALDH1A1 staining, and histological type in the different studies. On the other hand, ALDH1 high activity by Aldeflour assay is mainly correlated with poor prognosis. Because Aldeflour assay detects several ALDH1 isoforms (ALDH1A1, ALDH1A2, and ALDH1A3), we cannot rule out the role of other isoforms, i.e. ALDH1A2 and ALDH1A3. Recently, it has been reported that ALDH1A3 in particular contributes to Aldeflour high activity, which may be tissue and cancer specific, in murine HSC, murine pancreatic progenitor cells, and human breast CSCs [32]. Thus, we should be careful in reviewing AlDH1A1 overexpression and high activity in various cancer types.

#### Liver cancer

The biological effect of ALDH1 in the growth of hepatocellular carcinoma (HCC) cells and the maintenance of stem cell-like features in HCC remains unclear. In a previous study, our group [68] investigated the relationship between ALDH1A1 and clinicopathologic features in primary HCC surgical sections using IHC and qRT-PCR, and examined whether ALDH1A1 is an accurate CSC marker in HCC. The study found that ALDH1A1 was not a CSC marker in HCC, but that it did have the potential to serve as a therapeutic target in HCC.

Tanaka *et al.* [68] defined ALDH1A1-overexpressing cells as more intensely stained cells, compared with perivascular hepatocytes, which show moderately strong expression in the surrounding normal liver tissue. ALDH1A1 was expressed very heterogeneously and non-uniformly within the tumor tissue of HCC specimens. It is not clear whether this definition of ALDH1A1-overexpressing cells is equivalent to “ALDHbri cells” [33], which have been found in cancer tissues including breast, liver, and colon and in acute myelogenous leukemia, and are regarded as CSCs based on their proliferation rates, migration, and adhesion ability. Moreover, the metastatic potential of ALDHbri cells is greater than that of ALDH low cells, and ALDHbri cells contribute to cancer chemoresistance.

Likewise, previous reports on the liver suggest that high ALDH activity evaluated using flow cytometry could be a marker of liver progenitor cells in normal liver [69] and CSCs in HCC [70]. Additionally, ALDHbri cells are identified using the Aldefluor assay based on the enzymatic activity, which is attributed to ALDH1A1 expression. Meanwhile, ALDH1A1-overexpressing cells are identified using IHC based on the localization of ALDH1A1. Thus, ALDH1A1-overexpressing cells are considered to be different from ALDHbri cells in HCC.

In a separate study, Suzuki *et al.* [71] used IHC to evaluate ALDH1A1 in primary HCC specimens. After evaluating the percentage of ALDH1A1-overexpressing cells, ALDH1A1-high HCC was significantly associated with low serum levels of alpha-fetoprotein, well-differentiated pathology, and a favorable clinical outcome, in agreement with previous reports [68].

Additionally, Tanaka *et al.* [68] investigated the co-localization of ALDH1A1 with several CSC/progenitor markers (EpCAM, BMI1, CD13, CD24, CD90 and CD133) [70, 72-77] to evaluate stemness in ALDH1A1-overexpressing cells. In conflict with a previous report [70], the ALDH1A1-overexpressing cells did not exhibit co-expression with any of these CSC markers. Considering that the presence of CSCs is generally associated with poor histopathological grade and worse survival [78], the results suggest that ALDH1A1 is not a reliable CSC marker in HCC.

As a result, in HCC, the presence of a high percentage of ALDH1A1-overexpressing cells could be a factor indicative of well-differentiated pathology and favorable clinical prognosis. Furthermore, ALDH1A1-overexpressing cells appear to function as a differentiation marker rather than as a CSC marker in HCC.

#### Lung cancer

Increased ALDH1A1 expression was associated with poor survival in a cohort of non-small cell lung cancer (NSCLC) patients [55, 79, 80]. Gao *et al.* [81] reported that positive ALDH1A1 staining was detected in 41.28% (45/109) of the cases and ALDH1A1 mRNA expression was markedly elevated in most tumor tissues compared with adjacent normal tissues. Furthermore, higher ALDH1A1 expression levels were associated with a higher stage of disease (stage III+IV) and poor survival [81].

Jiang *et al.* [55] showed that the ALDH1A1-positive lung cancer cells could generate tumors *in vivo*. The expression of ALDH1A1 was positively correlated with the stage and grade of lung tumors and related to a poor prognosis for patients with early-stage lung cancer, which suggested that ALDH1A1 could be a potential prognostic factor and therapeutic target for the treatment of patients with lung cancer. However, Dimou *et al.* [82] reported contradictory results, indicating that ALDH1A1-negative expression in lung cancer patients corresponded to shorter survival compared with those with ALDH1A1-positive expression and that ALDH1A1 overexpression was associated with a favorable outcome.

A recent meta-analysis shows that increased ALDH1A1 expression is associated with poor overall survival and disease free survival in lung cancer patients [83]. ALDH1A1 may provide a therapeutic target for developing specific drugs to effectively eradicate lung CSC population and could potentially yield efficient therapeutic approaches for the treatment of lung cancer.

#### Ovarian cancer

The ALDH1A1-positive cell subpopulation has been demonstrated to be associated with chemoresistance in ovarian cancer patients [33, 84]. Meng *et al.* [85] reported that the stable knockdown of ALDH1A1 dramatically decreased the ability of ovarian cancer cells to form colonies. However, although ALDH+ cells detected using the Aldefluor assay demonstrated increased invasive properties compared with ALDH− cells, a difference in the invasive potential of a single isozyme ALDH1A1 was not seen. Nonetheless, ALDH overexpression is associated with many properties of ovarian cancer stem-like cells, such as enhanced invasion, colony formation, and chemoresistance [86]. ALDH1A1 plays a key role in the maintenance of ovarian cancer stem cell-like properties and might mediate carboplatin resistance [44, 67] through altered regulation of the cell cycle and DNA repair networks. However, increased expression of ALDH1 in ovarian cancer correlates with more favorable disease-free and overall survival [67].

#### Esophageal cancer (squamous cell carcinoma)

ALDH1A1 was not detected in normal esophageal epithelia, but it was found to be present at low levels in dysplastic basal cells [87]. The cytoplasmic ALDH1A1 was elevated in esophageal cells with increasing degrees of dysplasia and in carcinoma *in situ*. Additionally, ALDH1A1-high esophageal squamous cell carcinoma cells possess CSC properties and the expression of ALDH1A1 is associated with esophageal squamous dysplasia and carcinoma [87]. Yang *et al.* [87] detected the ALDH1A1 protein mainly in the cytoplasm of precancerous and cancer cells of the esophagus, consistent with reports on breast and ovarian tumors [44, 67]. Furthermore, in low-grade esophageal squamous dysplasia, ALDH1A1-positive cells were distributed in the basal layer of the mucosa, suggesting normal mucosal stem cells as the source of cancer stem-like cells [49].

ALDH1A1 expression also positively correlated with the Union for International Cancer Control stages, invasion depth, and lymph node metastasis of esophageal squamous cell carcinomas and was associated with shorter survival of patients. Thus, ALDH1A1-positive cells were preferentially distributed in the invasion frontier of esophageal squamous cell carcinomas and metastatic lesions [87]. Taken together, ALDH1A1-expressing cells are crucial for the development and progression of esophageal squamous cell carcinomas and ALDH1A1 may be used as a predictor of patient prognosis and a biomarker for malignancy of esophageal squamous cell carcinomas.

#### Stomach cancer

Li *et al.* [88] reported that ALDH1A1 was significantly associated with depth of invasion, lymph node metastasis, and stage of disease. In addition, survival times (overall survival and recurrence-free survival) of gastric cancer patients with high ALDH1A1 expression were significantly shorter than for those with low ALDH1A1 expression. Similarly, Wakamatsu *et al.* [89] revealed that ALDH1 is overexpressed in gastric cancer and is positively correlated with depth of invasion and TNM stage. Moreover, ALDH1 expression was significantly higher in diffuse-type lymph node metastasis than in the primary tumor, and ALDH1A1 was found to be overexpressed in highly invasive tumors, especially in T3 and T4 carcinomas [89]. As far as lymph node status was concerned, patients with lymph node metastasis tended to show elevated ALDH1A1 expression. Collectively, the strong association of high ALDH1A1 expression with gastric cancer aggressiveness suggests that ALDH1A1 could be a feasible target for cancer therapy [88].

#### Pancreatic cancer

Recently, increased expression of ALDH1A1 in a pancreatic cancer tissue microarray has been described, and was reported to correlate with a dismal prognosis [90]. Conversely, using immunohistochemical analysis on whole-mounted tissue slides, Kahlert *et al.* [91] demonstrated that low expression of ALDH1A1 is an independent prognostic marker for shortened disease-free and overall survival in ductal adenocarcinoma of the pancreas. These results are conflicting, but it is worth noting that the evaluation of ALDH1A1 expression was analyzed using different methodologies, which may explain these opposing results. In fact, by evaluating whole-mounted tissue slides, Kahlert *et al.* [91] found ALDH1A1 to be expressed heterogeneously within the tumor bulk, but concluded that a much higher fraction (74%) of the tumor specimen was positive, whereas Rasheed *et al.* [90] claimed that only 34% of the immunostained tumor samples were positive. Hence, using only 0.6-mm random tissue samples from morphologically representative tissue areas might obscure essential findings and result in an increased rate of false-negative results.

Kahlert *et al.* [91] also describe low expression of ALDH1A1 on whole-mounted tissue slides as an independent prognostic marker for a poor clinical outcome in pancreatic cancer, possibly because of the small number of patients in this subgroup. These data conflict with a previous report, which claimed that increased expression of ALDH1A1 was an adverse prognostic marker in a retrospective study. Therefore, to evaluate the role of ALDH1A1 as a prognostic and predictive marker for tumor progression and response to chemotherapy in pancreatic cancer, standardized prospective studies with a larger number of patients are required.

#### Breast cancer

Charafe-Jauffret *et al.* [92] reported that ALDH1A1-positive breast cancer cells are able to promote tumor invasion *in vitro* and promote tumor metastasis in mouse xenografts. Moreover, ALDH1A1 expression was an independent predictive factor for early metastasis and decreased survival in inflammatory breast cancer.

Ginestier *et al.* [44] reported that high expression of ALDH1A1 mRNA was correlated with poorer overall survival in breast cancer patients, and as a result, ALDH1A1 was the only ALDH1 isozyme capable of serving as a biomarker for predicting poor survival in breast cancer patients [93]. ALDH1A1 might be a major contributor to ALDH1 activity in breast cancer because only high expression of ALDH1A1 mRNA was found to be significantly correlated with poor overall survival in breast cancer patients. Thus, positive ALDH1 expression seems to be a predictive marker. Further, a recent meta-analysis indicates that ALDH1A1 can be used as an indicator of poor prognosis in breast cancer patients [94]. In breast cancer, ALDH1A1 expression is a good CSC marker and an important predictor of progression and poor survival.

#### Colorectal cancer

Surprisingly, overexpression of ALDH1 in colorectal cancer is not related to differences in survival [95]. Additionally, neither cytoplasmic nor stromal expression of ALDH1A1 was associated with prognosis in colon or rectal cancer [96], but a small proportion of colon cancer samples were discovered to be positive for nuclear staining of ALDH1A1. Furthermore, nuclear staining of ALDH1A1 in colon cancer was associated with a dismal prognosis. However, owing to the small number of patients showing nuclear expression of ALDH1A1, Kahlert *et al.* [97] did not consider this staining to be a useful prognostic biomarker for clinical outcome. Nevertheless, from a molecular viewpoint, this finding might be of certain interest with further experimental studies examining the molecular and biological function of ALDH1A1 [96].

Thus, ALDH1A1 is not a prognostic or predictive marker in colon or rectal cancers. However, considering that this is the first study to demonstrate that ALDH1 displays nuclear expression in a small subset of patients with colon and rectal cancers, it may be of importance for future studies. This is particularly encouraging because immunohistochemical analysis of ALDH1A1 expression in colon cancer is useful for the detection of nuclear expression in a small subpopulation of patients and is associated with shorter survival. Importantly, cytoplasmic expression is not clinically relevant as a prognostic or predictive marker in colorectal cancer [96].

### ALDH1A1 as a therapeutic target

Of the 19 known human ALDH enzymes, only a few have been characterized biochemically, specifically ALDH1A1, ALDH1B1, ALDH2, ALDH3A1, ALDH3B1 and ALDH7A1. Although these ALDH isozymes exhibit distinct substrate specificity, they also show an overlapping spectrum of substrates, making it difficult to precisely delineate isozyme-specific effects. Only three ALDH isozymes, ALDH1A1, ALDH2 and ALDH3A1, have been studied with respect to pharmacological inhibition. These are the enzymes involved in the metabolism of alcohol (ALDH2) and the anticancer oxazaphosphorine drugs, such as cyclophosphamide and procarbazine (ALDH1A1 and ALDH3A1) [98].

No antagonists have been developed that are specific inhibitors of the different ALDH isozymes. This lack of selectivity of available ALDH isozyme antagonists that have been tested as anti-cancer agents in the clinical setting has resulted in an unacceptable side-effect profile. Furthermore, the targeting of ALDHs requires careful attention to the delivery strategy, otherwise off-target toxicities are to be expected. However, Condello *et al.* [99] recently reported that an ALDH1A1-specific inhibitor was used to block ovarian cancer cell proliferation and survival. Further, the ALDH1A1 isotype positive subpopulation is related to chemoresistance [62, 85]. Thus, currently, an ALDH1A1-specific therapy with SC-signaling pathway inhibitors and/or antibody-based therapy is expected for targeting CSC.

In several recent studies, ALDH1A1 distribution patterns in normal tissues were distinct, and were classified into three types: 1) tissues with absent or limited ALDH1A1 expression (i.e., breast, lung and esophagus); 2) tissues with relatively weak ALDH1A1 expression (i.e., colon and stomach epithelium); and 3) tissues with extensive and high ALDH1 expression (i.e., liver and pancreas) [27, 47, 68, 90, 91]. ALDH1A1 can be effectively used as a CSC marker in tissue types that normally do not express ALDH1A1 at a high level (e.g., breast, lung, colon and stomach epithelium); however, it should not be used as a CSC marker in tissue types that normally express a high level of ALDH1A1 (e.g., liver and pancreas).

Overall, ALDH1A1-specific targeted therapy might be useful in cancer treatment. Of interest, Aldh1a1−/− mice are viable. This suggests that ALDH1A1 inhibition might not damage normal tissue SCs in ALDH1A1 targeted therapy for CSC elimination.

## CONCLUSIONS

There is accumulating evidence, based on the Aldeflour assay and IHC with isotype-specific antibodies, that supports the role for ALDH1A1 in SCs and CSCs. ALDH1A1 is considered a marker for these cells and may play a functional role in terms of self-protection, differentiation, and expansion of the SC populations. The modulation of ALDH1A1 might also play a key role in the regulation of growth and differentiation of both normal and cancer cells, also influencing some aspects of the cancer phenotype and prognosis.

ALDH1 and specifically its isotype ALDH1A1 can be useful as a CSC therapeutic target in cancer tissue types that normally do not express high levels of ALDH1A1, such as breast, lung, esophagus, colon, and stomach epithelium. However, for future cancer treatment, further studies are needed that identify specific ALDH1A1 inhibitors or inhibitors of other ALDH involved in CSC regulation without off-target toxicity. To maximize the efficacy of therapeutics, the contribution of the additional isotypes will need to be defined with additional studies. However, recently, a specific ALDH1A1 inhibitor has been developed for CSC target therapy. Future studies should investigate the key signaling pathways that regulate cancer-associated ALDH1A1 or expression of other ALDH isozymes in various types of cancers.
